# The effect of a limited number of projections and reconstruction algorithms on the image quality of megavoltage digital tomosynthesis

**DOI:** 10.1120/jacmp.v10i3.2970

**Published:** 2009-05-28

**Authors:** Vikren Sarkar, Chengyu Shi, Prema Rassiah‐Szegedi, Aidnag Diaz, Tony Eng, Niko Papanikolaou

**Affiliations:** ^1^ Division of Medical Physics CTRC at the University of Texas Health Science Center at San Antonio San Antonio TX USA; ^2^ Department of Radiation Oncology CTRC at the University of Texas Health Science Center at San Antonio San Antonio TX USA; ^3^ Department of Radiation Oncology University of Utah Salt Lake City UT USA

**Keywords:** megavoltage, tomosynthesis, algorithm, image quality

## Abstract

In order to investigate the effect of the number of projections on digital tomosynthesis image quality, images were acquired over a 40 degree arc and sampled into sets of 2 to 41 projections used as input to three different reconstruction algorithms: the shift‐and‐add, the Feldkamp‐Davis‐Kress filtered back projection algorithms, and the simultaneous algebraic reconstruction technique. The variation of several image characteristics, such as in‐plane resolution, contrast to noise ratio, artifact spread, volumetric accuracy, and dose, are investigated based on the reconstruction algorithms used and also the number of projections used as source data. The results suggest that only 11 projections are required since the various parameters checked do not improve much past that number. As a reconstruction algorithm, SART did best but took much longer to reconstruct images. Thus, if reconstruction time is a determining factor, filtered back‐projection looks like a better compromise.

PACS number: 87.57.C‐, 87.57.nf, 87.57.Q‐, 87.59.‐e

## I. INTRODUCTION

With the advent of Intensity Modulated Radiation Therapy (IMRT) and the ability to produce dose distributions that are highly conformal to the tumor volume, correct patient positioning becomes of prime importance. Solutions to this problem range from invasive techniques where markers or radio frequency beacons are directly placed inside the tumor, to non‐invasive techniques using ionizing radiation as is the case with computed tomography (CT) or using non‐ionizing radiation as exemplified by the use of ultrasound imaging.^(1)^ The increasing use of imaging techniques for the purpose of positioning the patient prior to radiation delivery has given rise to the field of Image Guided Radiotherapy (IGRT).^(^ ^2^ ^–^ ^6^ ^)^ With the recent developments in electronic portal imaging devices (EPIDs), there has been much work recently published in the field of cone beam CT^(^ ^7^ ^–^ ^10^ ^)^ using either the actual treatment beam or a kilovoltage (kV) beam from an X‐ray tube attached to the gantry. While a lot of work has been done with modified linear accelerators or high efficiency receptors to obtain all the projection images required for a complete CT reconstruction,^(10)^ the implementation of this technique depends on specialized equipment, which can involve relatively high costs. For a clinic with a conventional linear accelerator not optimized for imaging purposes, the operation of the machine in clinical mode only offers the possibility of programming one monitor unit (MU) or more. Thus, in these cases, each image can be acquired using the minimum of one MU. It is true that various groups^(^ ^9^ ^,^ ^11^ ^)^ have shown that images can be acquired with very low doses, but the machine used by these authors had specialized imaging equipment solutions, optimized for imaging at low doses. The present work uses a linear accelerator without such specialized equipment. If one MU is needed for each image, the dose that would be given to the patient for the acquisition of the approximately 100–200 projection images required for a full CT reconstruction becomes too large to be acceptable. The aim of this work is to explore the use of a reconstructed image set which will mimic a CT dataset while only requiring a fraction of the images needed for CT reconstruction.

The technique of tomosynthesis was first introduced in 1932 by Ziedses des Plantes.^(12)^ Its main advantage is that it uses only a subset of the projections required for CT reconstruction to retroactively produce any number of tomograms. Since only a subset of images is required, the imaging dose given to the patient is a fraction of what is given during imaging for CT reconstruction. Also, the images can be acquired faster, which usually means there is less chance for motion artifacts to be included in the final tomosynthesis image set. It is only recently that the technique has been applied to patient localization in radiotherapy.^(^ ^13^ ^–^ ^15^ ^)^ However, most of this effort concentrates on using data from a kV beam, and only a recent few publications^(^ ^16^ ^,^ ^17^ ^)^ look at the use of mega‐voltage (MV) images for the purposes of digital tomosynthesis. The use of the treatment beam and existing digital receptors to image the patient offers the main advantage that there is no need for further equipment purchase, as would be the case when using kV images that need a kV unit and an additional image receptor. While the two recent publications on MV‐based digital tomosynthesis looked at only one reconstruction algorithm – namely, filtered back‐projection based on the Feldkamp‐Davis‐Kress(FDK) methodology^(18)^ – this project investigates the effect of three different reconstruction algorithms on the spatial resolution, contrast to noise ratio, extent of spread of artifacts, and the volumetric accuracy of reconstructed objects from datasets reconstructed using the technique of megavoltage cone beam digital tomosynthesis (MV‐CBDT). The three techniques investigated are: (a) the shift‐and‐add (SAA) algorithm,^(19)^ (b) the simultaneous algebraic reconstruction technique (SART),^(20)^ and (c) the FDK algorithm. Although such comparisons have been done previously^(^ ^21^ ^,^ ^22^ ^)^ for kV images, the inherent low contrast of MV images warrants this investigation since the results may change due to the quality of the source images. Whereas the present work repeats a small part of the investigation by Descovich et al.,^(16)^ it also investigates three algorithms and looks at the effect of the number of projections used for a given tomosynthesis angle (40°) on the quality of the data sets produced by each algorithm.

## II. MATERIALS AND METHODS

### A. SAA algorithm

**Figure 1:**
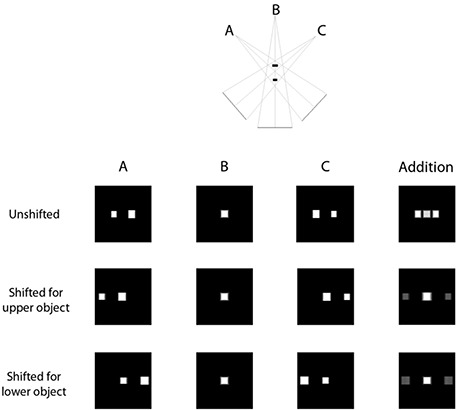
Illustration of the shift and add process.

This algorithm works by using geometry to line up projection images taken at different angles such that objects on the plane of reconstruction become more prominent while out‐of‐plane objects are smeared out. Objects at different heights above and below the isocenter will cast shadows at different parts of the detector, depending on what angle is used for image acquisition. Therefore, with prior knowledge of the angle at which the projection is taken, and depending on the height of the reconstruction plane relative to the isocenter location, each pixel is shifted by an appropriate amount. This is diagrammatically represented in Fig. 1.

As shown on the top row of images, the simple addition of the three acquired images produces an overlay of structures. However, once the appropriate shifts have been applied to each image, addition of the resulting images give rise to tomogram showing objects present at that level in high contrast whereas the out‐of‐plane objects, while still present, show up with much lower contrast.

The algorithm developed by Kolitsi et al.^(19)^ (1992) is a two‐step process. First, each image is transformed into a plane that is parallel to the plane of reconstruction (for our case, this is a horizontal plane). This is done by applying Eq. (1):

(1) $$h=\frac{id}{d\cos\alpha -i\sin\alpha }$$

where *h* is the distance of the particular pixel from the origin (at the center of the image) in the reconstructed plane, *i* is the distance from the origin in the receptor plane, *d* is the source‐to‐image receptor distance, and α is the angle at which the projection is obtained. The second step shifts each pixel by an amount

(2) $$r=\left(h-\frac{ad\sin\alpha }{b\cos\alpha -a}\right)\left(1-\frac{a}{b\cos\alpha }\right)$$

where a is the distance of the level of reconstruction above the isocenter and b is the source to isocenter distance.

Once all images have been shifted, they are added and normalized to give the final tomogram at level *a.*

### B. FDK algorithm

This is one version of the filtered back projection algorithm that is widely used for CT reconstruction. The algorithm was originally proposed by Feldkamp, Davis and Kress^(18)^ in 1984 and has been widely adopted for cone beam reconstructions, even though the algorithm produces an approximate solution. Only the central slice of the reconstructed object has an exact solution, based on a fan beam reconstruction. However, for small to moderate cone angles, the approximation works well. The general equation for reconstruction is

(3) $$g(x,y,z)=\frac{1}{2}\int_{0}^{2\pi }d\beta \int_{-\infty }^{\infty }\frac{D^{2}}{{\left(D-s\right)}^{2}}R(p,\zeta ,\beta )h(\frac{Dt}{D-s}-p)\frac{D}{\sqrt{D^{2}+p^{2}+\zeta^{2}}}dp$$

where *g(x,y,z*) is the value of the object at location (x,y,z), β is the angle relative to the z‐axis, *D* is the source to isocenter distance, R(p,ζ,β) is the projection data acquired at angle β,t=xcosβ+ysinβ,s=ycosβ−xsinβ, and (p,ζ) represents the coordinate system of the detector. The algorithm works in three steps:

1. First, the projection data is scaled as if it were measured at the plane containing isocenter.
2. Second, each row of each projection is individually filtered. Typically, a ramp filter (in frequency domain) is used to remove the radial blurring that occurs during the back projection process. The filtering is usually done in the frequency domain where the process involves multiplication of two functions rather than the computationally expensive convolution process that would be required in the Cartesian domain. For this project, a Hamming window was used to filter the images.
3. Finally, the filtered and weighted data is back‐projected over a grid to reconstruct the object.

For a complete mathematical description of the algorithm, refer to Feldkamp, Davis and Kress.^(18)^

### C. SART algorithm

This is a member of the family of algebraic reconstruction techniques which uses an iterative method to solve a large system of linear equations. The simultaneous algebraic reconstruction technique is one that updates the value of each voxel one projection at a time. Each voxel of the object to be reconstructed is assigned an initial value (zero in our case). A forward projection is performed and each voxel's value is proportional to the weighted difference between calculated and measured values of all pixels to which that particular voxel contributes. The general equation for the algorithm is

(4) $$v_{j}^{k}=v_{j}^{k-1}+\lambda \frac{\sum_{i}\left(\frac{p_{i}-\sum_{l}\omega_{il}v_{l}^{k-1}}{\sum_{l}\omega_{il}}\right)\omega_{\text{ij}}}{\sum_{i}\omega_{\text{ij}}}$$

where *j* represents the index of the voxel *v* under consideration, *k* is the iteration number, $\omega_{\text{ij}}$ is the contribution of voxel *j* to pixel *i*, and Λ is a constant of proportionality called the relaxation parameter, usually chosen to be between 0 and 1, with most studies choosing it to be closer to zero than unity. In this study, the relaxation parameter was set to 0.2. For this project, one iteration was defined to be complete when all projected images acquired had been processed, with the object updated at each step. Thus, iterations were stopped after a preset number of iterations, rather than quantifying the difference between projected data and measured data and stopping once that difference became less than a preset value.

### D. Image acquisition and processing

For all experiments using portal images, the images were acquired using an add‐on CCD camera EPID (Theraview Inc., Leusden, The Netherlands) mounted on a Varian clinac 600 C/D (Varian Oncology, Palo Alto, CA). According to the manufacturer's technical specifications, the images are acquired in a 512×512 matrix with 12 bit precision. The CCD camera is reported to have a dynamic range of 60 dB and a contrast detectability of at least 1%. For the purposes of this project, the following naming convention is used to denote gantry angles: the zero position (0°) is when the gantry is positioned for an anterior‐posterior field. Positive numbers are used for angles measured clockwise from the zero position, while negative numbers are used for angles measured counterclockwise from the zero position.

Based on the investigation of various tomosynthesis angles reported before in literature, a middle value of 40 degrees was chosen for this study. A total of 41 projection images were acquired every degree from −20° to 20°. With this acquisition geometry, the detector followed a circular path such that the distance between the source and imager was constant.

The images obtained were sampled such that anywhere from 2 to all 41 projections acquired were used as input to the three reconstruction algorithms. Table 1 summarizes which angles were used for each experiment.

**Table 1:** Image numbers used for each experiment.

*# Images Used*	*Angles used for reconstruction*
2	−20°,20°
3	Every 20 degrees from −20° to 20°
4	−20°,−7°,7°,20°
5	Every 10 degrees from −20° to 20°
6	Every 8 degrees from −20° to 20°
7	−20°,−14°,−7°,0°,7°,14°,20°
9	Every 5 degrees from −20° to 20°
11	Every 4 degrees from −20° to 20°
14	Every 3 degrees from −20° to 19°
21	Every 2 degrees from −20° to 20°
41	Every degree from −20° to 20°

Each algorithm was used to reconstruct the same number of slices (50) with a nominal slice separation of 5 mm. The in‐plane voxel dimension was set to 1 mm for the datasets reconstructed with the FDK algorithm and SART. The SAA technique uses the native pixel dimension of the EPID, which was 0.64 mm in this case. All source images were acquired with the EPID positioned 50 cm below the isocenter level. However, the images were resampled to obtain a 1 mm in‐plane voxel dimension for fair comparison with the other datasets reconstructed using the higher dimension.

### E. Spatial resolution

**Figure 2:**
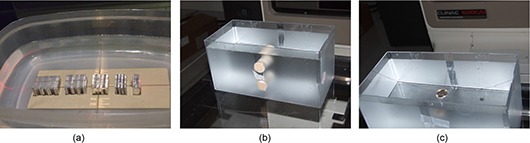
Phantoms used for various experiments. Fig. 2(a) spatial resolution test: (L – R) 5 mm to 1 mm patterns created by sandwiching appropriate number of 1 mm‐thick pieces of nylon ribbon between pieces of aluminum sheets of the appropriate thickness; (b) contrast resolution test: two cylinders of material with relative densities of $0.28\;g/{\text{cm}}^{3}$ (bottom) and $1.69\;g/{\text{cm}}^{3}$ (top) are positioned inside a water filled container with phantom set such that the cylinder's long axes are parallel to the gantry rotation axis and the isocenter lies between the two cylinders; (c) artifact spread investigation: a quarter suspended in water container so that isocenter is at the center of the coin.

An in‐house phantom was developed to visually assess the spatial resolution. Small sections (2.5cm×5cm) of aluminum and nylon sheets were used to create individual patterns representing patterns with one line pair every 2 mm, 4 mm, 6 mm, 8 mm and 10 mm. Five individual bar patterns were created, each containing four aluminum ‘bars’ separated by nylon ‘bars’ to create 3.5 line pairs per set. These blocks were placed on a block of Styrofoam and positioned in a water‐filled container and the phantom was positioned such that isocenter was at the center of the bar patterns as show in Fig. 2(a). EPID images were acquired as described above and CBDT datasets were reconstructed. These datasets were imported using ImageJ (National Institute of Health, Bethesda, MD) and the slice through isocenter was visually compared for each experiment.

Since the visual inspection is highly subjective, a second experiment was performed to measure the modulation transfer function (MTF) for each reconstructed dataset. The phantom in this case was a 6 mm‐thick slab of lead used to create a straight edge in the reconstructed datasets. The MTF was calculated as the Fourier transform of the gradient of a line profile taken through the edge.^(23)^ A total of five profiles were taken at different positions along the reconstructed image. Each profile was used to calculate an individual MTF curve and a sixth order polynomial function was fit to the data using Excel's curve fitting tool (Version 2007, Microsoft Corp, Redmond, WA). For the purposes of comparing the resolution for each dataset, the limiting spatial frequency was defined as that which creates an MTF of 0.2. The size of each bar in a pattern representing this spatial frequency was calculated and reported.

### F. Contrast

Two cylindrical inserts (Gammex RMI, Middleton, WI) with relative electron densities of $0.28\;g/{\text{cm}}^{3}$ and $1.69\;g/{\text{cm}}^{3}$ were positioned in a water‐filled container such that their long axis was parallel to the gantry's rotational axis. The phantom was positioned such that the isocenter was between the two cylinders as shown in Fig. 2(b). Images were acquired according to the protocol described before.

Datasets were reconstructed using all three algorithms and imported into ImageJ. A macro was created to use a $15\times 15\;{\text{mm}}^{2}$ region of interest (ROI) to sample the same region in the same slice for each dataset. For each cylindrical insert, the mean signal strength was extracted from the ROI placed in the middle of the cylinder. For water measurements, the ROI was placed in a region away from the cylinder location on the isocenter slice. The contrast was characterized by the signal difference to noise ratio (SDNR) defined as

(5) $$SDNR=\frac{\mu_{\text{object}}-\mu_{\text{water}}}{\sigma_{\text{water}}}$$

where $\mu_{\text{object}}$ is the mean signal in the ROI within the cylinder being considered, $\mu_{\text{water}}$ is the mean ROI value in the water region immediately adjacent to that cylinder and $\sigma_{\text{water}}$ is the standard deviation of the ROI in that water region.

### G. Artifact spread

Due to the use of projection images acquired over a limited angle, all CBDT reconstructions contain ghosting artifacts which are more pronounced in the reconstructed slices immediately adjacent to the plane on which the object is present. Wu et al.^(21)^ proposed the artifact spread function (ASF) as a metric to measure this effect. They defined the ASF as

(6) $$ASF(z)=\frac{{\bar{\mu }}_{Artifact}(z)-{\bar{\mu }}_{BG}(z)}{{\bar{\mu }}_{feature}(z_{0})-{\bar{\mu }}_{BG}(z_{0})}$$

where $z_{0}$ is the depth of the actual feature, *z* is the depth of an out‐of‐focus plane containing the artifact, ${\bar{\mu }}_{\text{Feature}}(z_{0})$ and ${\bar{\mu }}_{\text{BG}}(z_{0})$ are, respectively, the mean pixel value within the feature and in the background at depth $Z_{0}$, and ${\bar{\mu }}_{\text{Artifact}}(z)$ and ${\bar{\mu }}_{\text{BG}}(z_{0})$ are, respectively, the mean pixel value within the ghost image and background at depth *z*.

To test for the variation in ASF with depth, a quarter was suspended inside a water‐filled container which was positioned on the couch so that the coin was at isocenter level as shown in Fig. 2(c). Portal images were acquired and sampled as described before and objects were reconstructed using all three algorithms. The resulting datasets were imported into ImageJ and, since the signal due to ghost images appear at different locations on the different slices, a large area around the location of where the coin's ghost images are expected to lie was chosen as the ROI. The maximum pixel value within this ROI was chosen to be representative of the ghost image. This was repeated for five slices above and below the isocenter slice to determine the four parameters required to calculate ASF. A region well away from the coin was used to sample background and the median signal was chosen to represent the background signal. In this case, the maximum signal was not used since it would make the measurement sensitive to noise in the area.

### H. Volumetric accuracy

In order to check for volumetric accuracy, a $100\times 100\times 100\;{\text{mm}}^{3}$ virtual water phantom was created using MatLab (Version 8, The Mathworks Inc, Natick, MA), with voxel size of $1\times 1\times 1\;{\text{mm}}^{3}$. Within this phantom, a virtual cube, sphere, and cylinder of bony material were inserted. The phantom was imported into the Pinnacle treatment planning system (Phillips Medical Systems, Bothell, WA) as a CT set and the forward projection capabilities of the system was used to export digitally reconstructed radiographs (DRRs) every degree for gantry positions from −20° to 20°. The mean energy used for the DRR calculation was set to 2 MeV to ensure MV‐quality images. These DRRs were sampled as shown in Table 1 and datasets were reconstructed using the three algorithms. The reconstructed datasets were then imported back into Pinnacle and the contouring tool was used to contour each slice and determine the volume of each of the three inserted objects. The true volume was obtained by using the auto‐contour option on the virtual phantom itself.

### I. Calculation time

All calculations were performed on a dual‐core AMD Turion machine with a nominal processor speed of 1.9 GHz and 2 GB of RAM. All algorithms were programmed in‐house using Visual C++ (Version 5.0, Microsoft Corp, Redmond, WA). Table 2 shows the time required to calculate each dataset.

**Table 2:** Variation of reconstruction times with number of projections and algorithms.

	*SAA*	*FDK*	*SART (5 iterations*)
2 Projections	<1min	<1min	20 min
3 Projections	<1min	<1min	26 min
4 Projections	<1min	<1min	34 min
5 Projections	<1min	<1min	42 min
6 Projections	<1min	<1min	51 min
7 Projections	<1min	<1min	66 min
9 Projections	<1min	<1min	86 min
11 Projections	<1min	<1min	106 min
14 Projections	<1min	<1min	133 min
21 Projections	<1min	1 min	211 min
41 Projections	<1min	1.5 min	426 min

### J. dose measurements and calculations

To determine the dose delivered to different points during image acquisition, 3mm×3mm×1mm lithium fluoride (LiF) thermoluminescent dosimeters (TLDs) chips were positioned at different points within the Rando pelvic phantom (The Phantom Laboratory, Salem, NY). The TLDS were batched with responses within ±5%. The calibration was done by exposing one packet of TLDs to a known dose at the depth of maximum dose in solid water and then using that calibration factor to obtain the doses from the other packets used in the experiment.

Three points within the phantom were identified as locations of the bladder, prostate, and rectum. One packet of three TLDs was positioned at each point and images were acquired for 7, 11, and 14 projections over the 40° span. The minimum of 1 MU per image was used to acquire each projection. A CT study of the Rando phantom was also obtained and imported into Pinnacle3. The expected doses were calculated for the same three points using the treatment planning software and compared to the measured doses when these were available.

## III. RESULTS

### A. Spatial resolution

**Figure 3:**
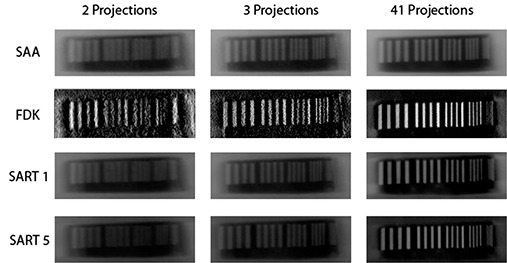
Tomosynthesis images depicting the isocenter slice through the resolution phantom.

**Figure 4:**
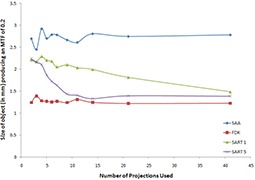
Variation of size of an object to create an MTF of 0.2 with reconstruction algorithm for different numbers of projection images used.

Figure 3 shows comparisons of a slice showing the bar pattern for a select few of the datasets generated. When two projections were used, only the 5 mm pattern was fully resolved, while three projections were enough to resolve all patterns except for the 1 mm model (which was not resolved even when all 41 projections were used). Figure 4 graphically shows the variation of object size for an MTF of 0.2 with reconstruction algorithm and number of projections used. The MTF values all confirm what was visually observed.

### B. contrast

**Figure 5:**
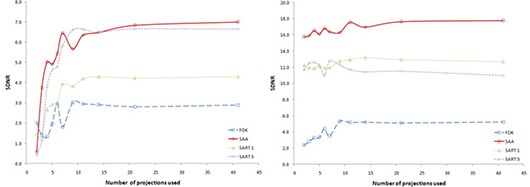
Variation of SDNR with number of projections used for reconstruction. Left curve shows behavior for central slice through bone insert (with a density of $1.69\;g/{\text{cm}}^{3}$); right curve shows behavior for central slice through lung insert (with a density of $0.28\;g/{\text{cm}}^{3}$).

Figure 5 shows the variation of SDNR with number of projections used and reconstruction algorithm for bone and lung. Since higher SDNR values correlate to higher contrast, the results show that the FDK algorithm does the worst for both cases, while the SAA algorithm produces either the best contrast or values comparable to the best. For all cases, the SDNR value increases when the number of projections used to generate the data is increased, until a plateau is reached.

### C. Artifact spread

**Figure 6:**
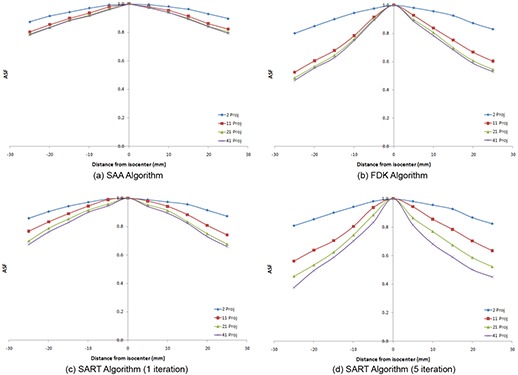
Representative graphs depicting the variation of ASF with number of projections used for each of the algorithms: (a) SAA algorithm; (b) FDK algorithm; (c) SART algorithm (1 iteration); (d) SART algorithm (5 iterations).

**Figure 7:**
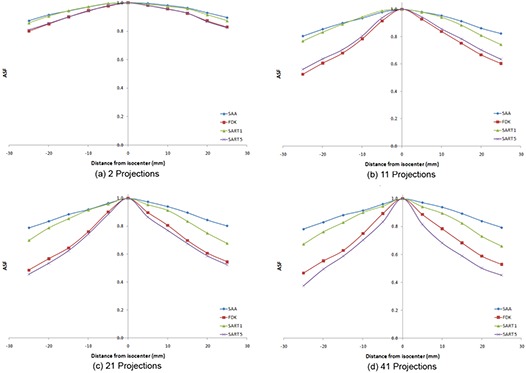
Variation of ASF with algorithm used for various numbers of projections: (a) 2 projections; (b) 11 projections; (c) 21 projections; (d) 41 projections.

Figure 6 shows the variation of ASF with number of projection used for each algorithm, while Fig. 7 shows the variation of ASF with the algorithm used when a particular number of projections were used. Since the object used in the study has thickness much smaller than the reconstruction slice separation, the ideal ASF curve would be a delta function showing that the signal from a particular object is only present on one slice. However, since only a limited scan angle is used in DTS, there are always some ghost signals that are present on some adjacent slices, making the ASF curves broaden. From the tests in the study it was concluded that, in general, SAA performs the worst; for lower number of projections, FDK performs best. When more than 21 projections are used, SART performed best when run for 5 iterations. In general, increasing the number of projections used lead to a decrease in ASF.

### D. Volumetric accuracy

**Figure 8:**
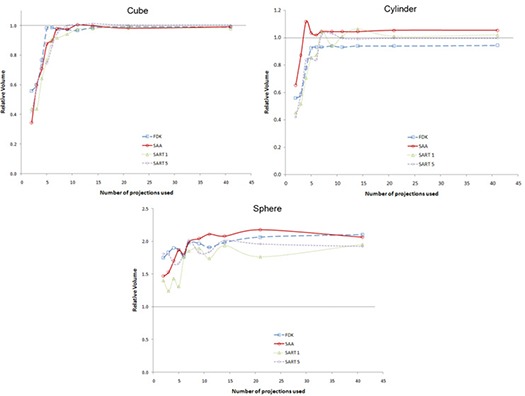
Variation of relative calculated volume with number of projection used for different algorithms: cube (top left curve); cylinder (top right curve); sphere (bottom curve).

Figure 8 shows the variation of calculated volumes of the three embedded objects with number of projections used for each algorithm. The horizontal line denotes the “true” volume as determined using the auto‐contour algorithm on the phantom. For all four algorithms, the calculated volume of the cube and cylinder quickly converges towards the actual value. For the sphere, the calculated value converges towards a value that is approximately twice that of the true value.

### E. dose measurements and calculations

**Figure 9:**
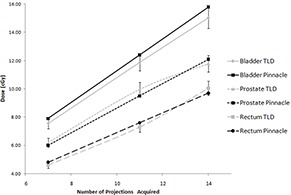
Comparison of dose measurements from TLDs and Pinnacle.

Figure 9 shows a plot of the doses as measured using TLDs and calculated in Pinnacle3. The error bars on the TLD measurements reflect the 5% acceptance criterion used while batching our TLDs. Table 3 shows the doses at the three sites as calculated using Pinnacle3.

**Table 3:** Variation of pinnacle calculated dose with number of projections used.

*No. of Projections Used*	*Bladder Dose (cGy)*	*Prostate Dose (cGy)*	*Rectal Dose (cGy)*
2	2.2	1.7	1.4
3	3.4	2.6	2.0
4	4.5	3.4	2.7
5	5.6	4.3	3.4
6	6.7	5.1	4.1
7	7.9	6.0	4.8
9	10.1	7.7	6.2
11	12.4	9.5	7.6
14	15.8	12.1	9.7
21	23.5	17.9	14.3
41	45.9	35.0	28.0
Portfilm^a^	12.5	11.2	7.3

^a^ “Portfilm” doses were calculated using the protocol clinically used to acquire weekly portfilms at the clinic for prostate cases; 6 MU are used to acquire an AP projection and 8 MU are used to acquire the lateral projection, for a total of 14 MU per image set.

## IV. DISCUSSION

The spatial resolution experiment shows that the limiting resolving power of 2 mm is achieved almost immediately regardless of the algorithm used for reconstruction. It should be made clear that all of these resolution measurements are for the in‐plane measurements, since the out‐of‐plane resolution is expected to be worse for a limited‐angle scan. If only these results were considered, it seems like there is no advantage of any one algorithm over another once the minimum of three projections is met. However, combining the contrast to noise test, some algorithms start to stand out. From Fig. 3, it is clear that the FDK reconstructions are noisier than any of the other datasets. Also, it should be noted that the FDK algorithm basically acts as a high‐pass filter for in‐plane structures when used in limited‐angle scans (as is the case here). While this causes edges to be sharper and increases their visibility (see Fig. 3), it also leads to a large drop in contrast.^(24)^ It therefore comes as no surprise that the SDNR for FDK datasets are consistently lower than those produced by the other algorithms. Since the SAA algorithm works by shifting the EPID images directly, the noise level of the datasets produced using that method is related to the amount of noise in the portals themselves. SAA also is the worst at removing artifacts, as shown by the ASF measurements. This makes the noise levels even lower due to the smearing from out‐of‐plane structures. As expected, the SAA algorithm produced high SDNR values throughout, which may lead to the wrong assumptions that this is the optimal reconstruction algorithm. However, the values are not due to higher contrast but are actually due to much lower noise levels. In fact, the SAA images have some of the worst contrast of all the reconstructions.

It is not surprising that ASF is worst for lower number of projections used as input data. It was already stated that the out‐of‐plane objects are still partially present in every slice. The ghost signals are actually smeared along the planes not containing the object. When only two projections are used, then the ghosts actually form two distorted images, a doublet. The ghost intensity is shared between these two artifacts. As more and more projections are used, the intensity is actually smeared across a larger area. Since ASF looks at a ratio of ghost contrast to object contrast, the ASF gets smaller in magnitude when more projections are used since contrast of the ghosts gets smaller.

**Figure 10:**
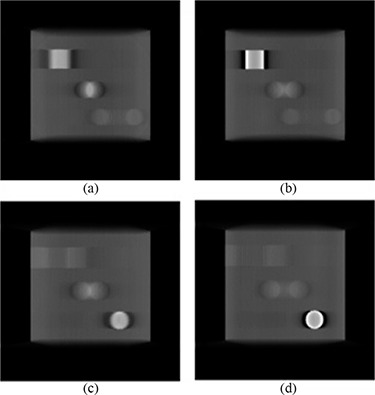
Slices through the SART reconstructed object showing: (a) absence of cube; (b) presence of cube; (c) absence of cylinder; (d) presence of cylinder.

The volumetric accuracy test showed that the calculated volumes always trended towards a constant value with an increasing number of projections used for the dataset calculation. For the two objects that showed constant cross‐sections in the DTS slices – namely the cube and cylinder – the calculated volumes got very close to the true value as the number of projections used increased. For objects with a varying cross‐section, the calculated values were always overestimated and, while there was convergence in volume, it was towards a value that was more than twice the true volume. With prior knowledge of what the objects looked like, it was easy to determine how to contour objects which had a constant cross‐section, and also to determine when the object was not actually present on a slice because the signal was only partially overlapping (see Fig. 10). For the datasets reconstructed using SART and FDK, a halo that appears immediately to the left and right of the high contrast objects greatly aided in the contouring.

**Figure 11:**
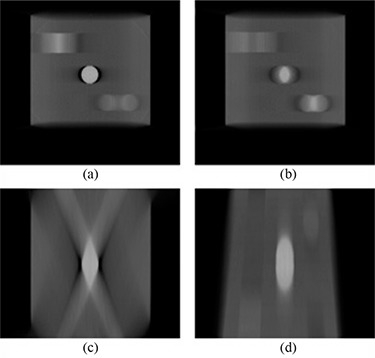
Slices through the SART reconstructed object: (a) coronal slice through center of sphere; (b) coronal slice through non‐central part of sphere; (c) axial slice through center of sphere; (d) sagittal slice through center of sphere.

However, for the spherical object, the situation is more complex. Unlike an object with constant cross‐section, incomplete overlap of signals does not mean absence of the object from the slice but, instead, may mean that the object is only partially present. Figure 11(a) shows the central slice through the sphere and represents the expected circular object. While one would expect to see smaller circles as one moves away from the central slice, what is instead observed are oval areas of overlap, as shown in Fig. 11(b). An explanation for this behavior is that projections were never taken at large angles. For example, if a projection was obtained at 90 degrees and showed a circular shape, the algorithms would then force the elongated region in off‐center slices through the sphere to be made smaller and more closely represent a circle rather than an oval. This behavior is also clear from the central axial (Fig. 11(c)) and sagittal (Fig. 11(d)) slices through the reconstructed sphere. Instead of a sphere, the object reconstructed looks more like an ovoid. This partially explains why an object with a larger volume is contoured. In terms of variation with number of projections, it seems that as few as 11 projections give rise to calculated volumes that are close to the ultimate volume obtained using all 41 projections.

**Figure 12:**
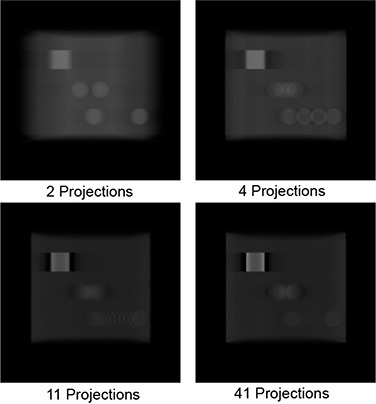
Corresponding slices showing the appearance of ghost images from the same object when (a) 2 projections, (b) 4 projections, (c) 11 projections and (d) 41 projections are used as source data.

An effect also visible in all reconstructions using very few projections is that the ghost images sometimes actually appear as a ringing artifact. This effect is illustrated in Fig. 12 which depicts the same slice from different reconstructions of the cube with objects used in the volumetric accuracy test. In extreme cases, such as in Fig. 12(a) where only two projections were used, the ghost images appear as the doublet previously mentioned. This is apparent in the middle part of the subfigure, where the signal from the sphere has separated. As the number of projections is increased, the effect becomes more of a smearing but other objects may show edges that appear to show the ringing effect. For example, in Fig. 12(c), the region where the sphere should be shows a more uniform smear but the bottom right region, which is meant to represent the cylindrical insert, shows the ringing effect. This is due to a stair‐step type of signal artifact that occurs as a result of the large angle between projections. This type of artifact is unfortunately something that cannot be filtered out, and users of CBDT image sets from under‐sampled image scans will have to expect this artifact and learn how to work around it.

Distortions in the shape of objects are inherent with the technique of digital tomosynthesis due to the limitation of imaging only over a short arc and never seeing the whole object. This, in turn, means that DTS‐based datasets may not be used for such tasks as treatment planning in their native form where a correct estimation of volumes is very important. However, the spatial accuracy of reconstructed images was also investigated. This was done by measuring the distance between the various bar patterns used in the spatial resolution phantom. When the image‐based distances were compared against those physical measurements, all agreed within 1 mm (or one pixel). This led to the conclusion that the reconstructions are accurate in terms of spatial accuracy.

The dose measurements show the expected increase in dose to all three points considered with an increasing number of projections used. When compared with the doses each point gets from the current portal imaging protocol, it is clear that approximately the same dose is delivered to acquire the portal image set as would be given during the acquisition of 11 projections.

## V. CONCLUSIONS

The purpose of this project was to determine the effect that the number of projection images used to reconstruct MV‐CBDT datasets had on various aspects of these reconstructions. The current imaging protocols for portal images gives the same amount of dose that would be given to acquire 11 projection images. Keeping this number of projections in mind, as far as the spatial resolution is concerned, all of the bar patterns are already resolved, except for the 1 mm pattern which never is. For all algorithms, the SDNR and reconstructed volume values seem to have already reached a plateau. ASF values will increase slightly when more projections are used. Since reconstruction times are linear with number of projections, the reconstruction times are on the order of less than 1 minute for SAA and FDK to 21 minutes for one iteration of SART.

The authors recognize that this evaluation is purely qualitative but also feel that, when all the tests are evaluated together, 11 projections seem like a good number of projections to aim for, especially because some of the tests – specifically, the SNR and volumetric accuracy investigations – seem to converge once this number of projections has been reached. For the ASF evaluation, there is a very small improvement when more than 11 projections are used but, given the increased dose with more projections, the authors do not believe the gain is worth the risk from increased radiation.

As it stands, the SAA algorithm does not look like a good candidate for reconstruction since artifact spread is so high compared to the other algorithms. While FDK produces noisy images, reconstruction times are much lower than SART, even for a single iteration. Both SART and FDK produce comparable artifact spread when 11 projections are used. The choice of algorithm between SART and FDK depends on the user. If this is to be clinically used, then the current reconstruction times for SART makes the algorithm impractical for the purposes of image reconstruction. Therefore, for clinical use, it would seem like FDK would be a better choice, if the end user is willing to live with the noisier images. The FDK images may be improved in certain cases where spatial resolution is not as important as contrast and a more aggressive filter is used for the purposes of noise removal. If, on the other hand, the problem of reconstruction times is solved either with better software implementation or with hardware acceleration, then SART would certainly be the algorithm of choice.

It should be pointed out that this study's conclusions are specific to digital tomosynthesis with projections acquired using a CCD‐based camera and based on the phantom geometries investigated. Artifact spread is a known problem with digital tomosynthesis and the contrast and resolution of images will be affected by out‐of‐plane structures, something that will change between subjects. However, this is the first study, to the best of the authors’ knowledge, investigating the effect of number of projections and reconstruction algorithm on the final quality of images for megavoltage digital tomosynthesis. The authors are confident that the conclusions, demonstrating that MV‐CBDT reconstruction does not require the use of projections acquired every degree, will remain valid for other phantom geometries and image acquisition equipment since most parameters end up reaching a plateau after a critical number of projections is used. Future work should repeat the current experiment while varying the tomosynthesis angle, and also investigate whether imagers with different sensitivities produce different results.
